# Patient-physician interaction in general practice and health inequalities in a multidisciplinary study: design, methods and feasibility in the French INTERMEDE study

**DOI:** 10.1186/1472-6963-9-66

**Published:** 2009-04-22

**Authors:** Michelle Kelly-Irving, Christine Rolland, Anissa Afrite, Chantal Cases, Paul Dourgnon, Pierre Lombrail, Jean Pascal, Thierry Lang

**Affiliations:** 1 UMR INSERM 558 Epidémiologie et analyses en santé publique, Faculté de medicine, F-31073 Toulouse, France; 2 IRDES, 75018 Paris, France; 3 Laboratoire de santé publique, CHU de Nantes, 44093 Nantes, France

## Abstract

**Background:**

The way in which patients and their doctors interact is a potentially important factor in optimal communication during consultations as well as treatment, compliance and follow-up care. The aim of this multidisciplinary study is to use both qualitative and quantitative methods to explore the 'black box' that is the interaction between the two parties during a general practice consultation, and to identify factors therein that may contribute to producing health inequalities. This paper outlines the original multidisciplinary methodology used, and the feasibility of this type of study.

**Methods and design:**

The study design combines methodologies on two separate samples in two phases. Firstly, a qualitative phase collected ethnographical and sociological data during consultation, followed by in-depth interviews with both patients and doctors independently. Secondly, a quantitative phase on a different sample of patients and physicians collected data via several questionnaires given to patients and doctors consisting of specific 'mirrored' questions asked post-consultation, as well as collecting information on patient and physician characteristics.

**Discussion:**

The design and methodology used in this study were both successfully implemented, and readily accepted by doctors and patients alike. This type of multidisciplinary study shows great potential in providing further knowledge into the role of patient/physician interaction and its influence on maintaining or producing health inequalities. The next challenge in this study will be implementing the multidisciplinary approach during the data analysis.

## Background

The interaction between patients and their general practitioner (GP) is a key element in the efficiency and usage of health services, and varies depending on patient characteristics [1]. The nature and quality of the relationship between patients and their physicians affects communication, medical advice, satisfaction, and diagnosis [2-5]. There is a rich literature on the interaction between patients and physicians wherein several models of interaction are observed [6]. In recent years and especially within the context of increasing chronic disease prevalence, and better access to information, the model typology has veered away from the paternalistic model, grounded in medical authority, towards more patient-centred models where the relationship is increasingly based on negotiation and cooperation [7,8]. It is therefore important to identify the main factors in the physician's response likely to affect their patient's subsequent health trajectory.

If, as evidence suggests, the patient-physician relationship does affect a patient's health care trajectory, how they are treated and their compliance with treatments, this could in turn lead to health inequalities which will permeate across the health care system. Bensing et al (2006) highlight that a patient's inclination to participate in medical decision making varies by characteristics such as age, gender, education, coping style, and severity of condition [9]. Street et al note the importance of a shared identity between patients and physicians facilitating more positive health care interactions [4,10]. Furthermore, they found that the way a physician perceives a patient (intelligent, compliant etc) affects how they treat them during the consultation. Gender and age are also important influences on the doctor-patient relationship, with more smoking and alcohol-related advice being given to men and older patients [1,3]. Doctors also communicate and treat their patients differently according to other social characteristics such as social position and ethnicity. Bao et al found that physicians were less likely to discuss cancer screening tests with patients who had a lower education level, and with patients from low income groups [11]. Physicians were less likely to initiate post-angiography discussions during consultations when the patient was black, and conversely, black patients were les likely to initiate this type of discussion with their doctor [12]. Black patients, and patients whose ethnicity was discordant with that of their physician were found to receive significantly less information compared to their white or racially concordant counterparts [13,14]. Further exploration into the black-box of patient-physician interaction may highlight how and where some aspects of health inequalities are produced, and indicate how changes can be made in general practice to reduce health care inequalities linked to patients' social characteristics.

The overall objective of this study is to ascertain the mechanisms at play and impact of the patient-physician interaction in general practice on health inequalities using approaches from several disciplines. The study aims to address the following questions: Does the type of care patients receive in general practice vary by their social characteristics and therefore contribute to the production of health inequalities? What are the key elements of patient-physician interaction in the production of these inequalities? Finally, can recommendations be made to physicians about their interaction with patients that may help reduce health inequalities?

The theme of nutrition, overweight and obesity was deemed of particular interest, allowing for an exploration of the research questions via a specific health topic. Overweight and obesity is a socially distributed phenomenon, with an inverse trend in obesity prevalence by household income [15] and similar graded associations found between body size and educational attainment [16]. As well as the public health issues linked with obesity as a risk factor for many chronic diseases, the social stigma of being overweight is an important factor that may influence how the subject is raised or dealt with during a consultation. This topic was chosen because the consultation could be analysed in terms of the diagnosis of a weight problem, as well as the preventative recommendations made by the GP and any treatments mentioned or prescribed. Furthermore, guidelines for good practice in the context of advice on nutrition have been made available by several French public health institutions. The effect of these recommendations on the patient, and non-adherence could also be highlighted and analysed in terms of potential links with health inequalities.

## Methods and design

As an interdisciplinary project, the INTERMEDE study consists of several disciplinary teams, epidemiologists, sociologists and economists, collaborating using qualitative and quantitative research methods to investigate the main study objectives. The study focuses on general practice surgeries located in three French districts: Ile de France (IF), Midi Pyrenées (MP) and Pays de la Loire (PL). The design has two main phases: a qualitative phase where researchers observed consultations and conducted post-consultation 'mirrored' interviews with patients and physicians separately; and a quantitative phase where data were collected using 'mirrored' questionnaires whereby patients and their GPs were asked the same questions respectively after the consultation. This paper will describe the design and methods used in this pilot study, and will discuss the feasibility of this type of interdisciplinary project.

### Study Design

The qualitative phase of the study is an ethnographic observational design followed by semi-structured interviews. The quantitative phase is a cross-sectional design using mirrored questionnaires completed by patients and GPs independently. Each phase was conducted independently of the other, on a separate sample of GPs and patients. The two phases occurred sequentially: first the qualitative phase was conducted from March to September 2006 followed by the quantitative phase in September and October 2007.

### Eligibiligy

The criteria for patient inclusion in both phases of the study were as follows:

• being aged over 18 years;

• not attending the GP's surgery in an emergency;

• not being a first-time patient;

• not being pregnant or having given birth in the last six months,

• giving informed consent to participate in the whole study.

### The qualitative phase

Data were collected via ethnographic observation of consultations where the researcher observed all verbal and non verbal communication during the consultation. After the consultation semi-structured interviews focussed on the consultation were conducted with the patient and the physician independently. Both the consultation and the interviews were audio-recorded.

### Sampling

A total of 11 GPs were included from the three districts, three in PL, five in MP and three in the IF all of whom had volunteered to participate after hearing about the study via several GP networks: the Toulouse Department for general medicine and the French society for general medicine. They did not receive any financial compensation for their participation. Their surgeries were located in town-centres, residential areas, as well as rural areas around three cities (Nantes, Toulouse and Paris) and they worked in groups or in individual practices. Among the 11 GPs observed, three were women. Table 1. shows the number of GPs and patients in the qualitative sample by site as well as the number of ineligible patients excluded and the number of those who refused to participate.

**Table 1:** Total sample of GPs and patients in qualitative phase by region

**Site**	**GPs (n)**	**Included patients (n)**	**Ineligible patients (n)**	**Refusals (n)**	**Total Patients contacted (n)**
Ile de France	3	17	142*	0	159
Midi Pyrenees	5	18	30	5	53
Pays de la Loire	3	13	22	2	37

**Total**	**11**	**48**	**194**	**7**	**249**

* In this area, ineligible patients and refusals have been not distinguished

In total, 249 consultations were observed by trained researchers and 48 were followed-up for separate interviews with the patient and GP, all of which have been transcribed. These were selected based on the specific health topic of nutrition and overweight/obesity. The patient was invited to be interviewed if they were overweight or obese (≥25 kg/m^2^) or if the subject of nutrition or weight management was discussed during the consultation, as well as if they fulfilled the eligibility criteria for the study. Among the 201 consultations not followed-up, 194 patients did not fulfil the eligibility criteria and 7 declined to participate further. A particularly large sample of patients were contacted in the Ile de France region (n = 159) versus those contacted in Midi Pyrenees (n = 53) or Pays de la Loire (n = 37). This is indicative of the greater difficulty in recruiting patients to the study in this region, where the capital, Paris, is located. Two sociologists and one anthropologist conducted the interviews, all of whom were women.

Among the 48 patients who were selected for the post-consultation interviews, 27 were women, the median age was 60, and 40 had a BMI ≥ 25. The body of data collected for each consultation therefore consisted of a) observational data from the consultation, b) an interview with the patient and c) an interview with the GP, together forming a complete monograph for each of the 48 consultations included in the qualitative phase.

### Data collection

The researcher was present during the consultation taking ethnographic notes, and the consultations were also recorded using an audio tape. The researchers observed all physical examinations that took place during the consultation, except for intimate gynaecological or urological examinations. For intimate examinations the researcher either left the consultation for the duration of the examination, returning afterwards, or remained present recording the discussion but not observing the examination itself. At the end of each observed consultation they conducted a debriefing interview with the GP noting: the sequence of events during the consultation, whether objectives were attained, and information adequately passed on as well as the quality of the relationship, all from the GP's perspective. After the consultation the researcher set a date to interview selected patients in their home asking them about the content and nature of the consultation, their relationship with the GP, information, treatment or advice they received during the consultation and whether they were satisfied with the consultation and any follow-up care.

### The quantitative phase

This cross-sectional quantitative phase of the study took place at the GP's office over a two week period between September and October 2007. The research assistants who collected the data attended a one-day training session in the week before the data collection began that aimed to harmonise their approaches and troubleshoot potential problems.

### Sampling

**Figure 1:**
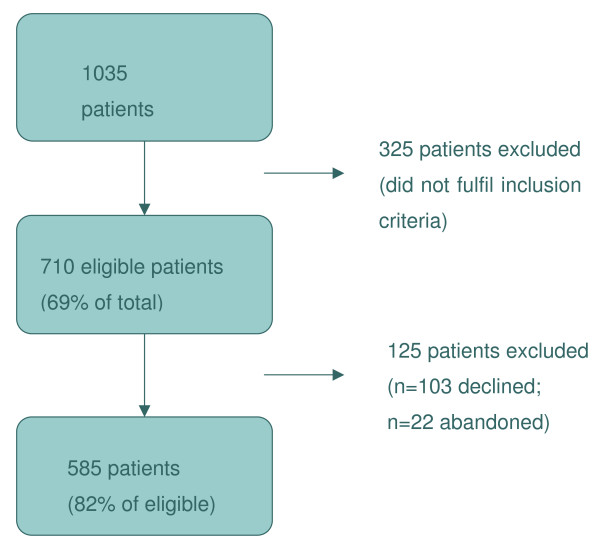
**Diagram of quantitative sample**.

Overall the sample consisted of 27 GPs from three French regions (IF, MP and PL) who were recruited on a volunteer basis via GP networks: the Toulouse Department for general medicine and the French society for general medicine. Though variations on the age, sex, seniority and type of practice are present within the group of GPs, they by no means represent the full spectrum of practicing physicians in urban France. In total 1035 patients were approached by the researchers in the different GP waiting rooms at the three sites (see Table 2). Among these 710 (69%) fulfilled the eligibility criteria for inclusion. By the end of the data collection period 125 individuals had either refused to participate (n = 103) or abandoned the study (n = 22). The final overall sample for the quantitative phase is therefore 585 patients, which represents 82% of the sample of eligible patients (see Figure 1).

**Table 2:** Total sample of GPs and patients in quantitative phase by region

**Site**	**GPs (n)**	**Patients (n)**
Ile de France	9	214
Midi Pyrenees	10	198
Pays de la Loire	8	173

**Total**	**27**	**585**

The patients were recruited over a two day period in the surgery waiting room where research assistants gave each visitor a preliminary questionnaire to complete. The first short questionnaire enabled the research assistant to establish whether the patient fulfilled the eligibility criteria for inclusion in the study. The questionnaire also allowed data to be collected on respondents who refused to participate. The patient and the GP's consent forms outlined that the general theme of the study was about patient-physician interaction. All data were anonymised, and the study received approval from the French Data Protection Authority [17].

### Data collection

The quantitative phase was a cross-sectional study of patient-physician interaction. The final sample consisted of 27 GPs and 585 patients. Data were collected via structured survey questionnaires completed by patients and their GPs. These consisted of three patient and two GP questionnaires:

#### Patient questionnaires

##### 1. The pre-consultation short questionnaire

This was a short preliminary questionnaire for self-completion by the patient with assistance from the research assistant where necessary. This was given to all patients in the waiting room eligible for participation in the study. It asked patients to report their age, gender, occupation, height, weight, perceived health and the reason for their visit to the GP's surgery. The research assistant was present when patients filled-in the questionnaires in case of disability, comprehension, language or literacy problems, and noted the reason for refusal where relevant.

##### 2. The post-consultation questionnaire I

This was a longer questionnaire completed face-to-face with the research assistant in a private room. It was completed immediately after the consultation and asked about the content of the consultation and patient satisfaction, more detailed questions about the patient's health status, and for information on treatments or prescriptions they were taking. The research assistant also measured and weighed the patient.

##### 3. The post-consultation questionnaire II

This questionnaire was completed over the phone by a trained research assistant two weeks after the initial consultation. It asked questions about the patient's compliance to treatments or recommendations and where relevant reasons for non-compliance, and it asked about the patient's opinions about their weight, nutrition and other health issues. Patients were also asked about their expectations of the patient/physician relationship.

#### GP Questionnaires

##### 1. The post-consultation questionnaire

This questionnaire mirrored many of the questions asked of the patient in their pre and post-consultation questionnaires. It was a self-administered questionnaire in CAPI (computer-assisted personal interviewing) format that was filled in by the GP after each consultation asking them to report on the nature of the consultation, any diagnoses or examinations that took place, any treatments prescribed, whether preventative advice was given. The GP also gave an assessment of the health status and personal characteristics of their patient.

##### 2. The general information questionnaire

This self-completion questionnaire was completed by each GP at the end of the study. The GP is asked to report their age, gender, seniority, height, weight, and other personal characteristics. They were also asked about their own views and values with regards to the practice of medicine. Some of the questions concerning opinions and beliefs were also mirrored in the patient questionnaires.

**Figure 2:**
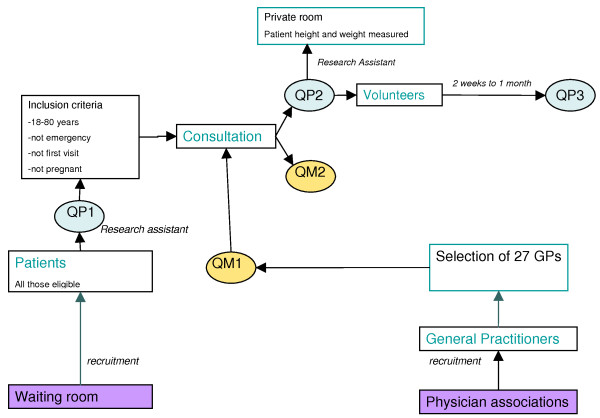
**Diagram of quantitative data collection procedure**. Patient questionnaires. QP1 – short pre-consultation questionnaire. QP2 – post-consultation questionnaire. QP3 – second post-consultation questionnaire 2 weeks to 1 month later. GP questionnaires. QM1 – general information on the GP. QM2 – post-consultation questionnaire.

Figure 2. explains the data-collection procedure for the quantitative phase, outlining the chain of events and when the various questionnaires were completed.

## Discussion

### Potential limitations

All studies are subject to bias and error caused by their inherent design, or by unforeseen methodological limitations. The INTERMEDE study is no exception, and the potential bias and errors that may be important are discussed below.

Selection bias is caused by the existence of systematic differences between those who take part in a study and those who do not ([18] p.166). This means that selection bias can cause a misrepresentation of the population within the study sample, and therefore potentially render results and findings invalid, as they do not represent the results that would have been found in the wider population. In this study selection bias may have been introduced at two points; first, at initial sampling, when individuals were assessed against the eligibility criteria, and second, when patients declined to participate or dropped out of the study. In the first instance, the patients could have been wrongly deemed eligible, or ineligible, and in the second instance, patients with specific characteristics could be systematically refusing to participate or dropping out, or conversely, patients with specific characteristics could be systematically agreeing to participate. These factors could potentially lead to selection bias, whereby the sample of patients was not representative of the 'real' body of patients due to the selection procedure. However, data were collected on patients who refused to participate and analyses will be conducted to determine if selection bias is likely to be present, and allow for results to be adjusted accordingly.

Selection bias could also be present among the sample of GPs who volunteered to participate. Bias could be introduced via two mechanisms, firstly through the baseline catchment of GPs contacted via the GP networks (the Toulouse Department for general medicine and the French society for general medicine), and secondly through the voluntary nature of their participation in the study. The GP networks could influence the types of individuals contacted. It is possible that certain types of GPs are more likely to be members of such organisations, and in this instance, many of the GPs who volunteered were implicated in teaching or training junior colleagues and had a specific interest in improving the quality of their role as community or family doctors. This could render the GP more likely to accept participation in a study, and mean that the GPs involved are more attentive to the quality or advancement of their work. Furthermore, the participants who all volunteered may have had a specific interest in this type of multidisciplinary study, or a special affinity for the subject matter of patient-physician interaction. These aspects of samples based on volunteers may also influence the types of GPs participating. Both potential type of selection bias could mean that the sample of GPs participating in the study is not a true representation of the GP population in France, and notably that GPs particularly interested in improving and advancing the nature of their work are over-represented in this study. These factors need to be kept in mind when interpreting results from the qualitative or quantitative analyses.

Knowledge and awareness that the study was on the patient-physician interaction could influence patients' as well as GPs' behaviours in several ways. It could determine a patient's decision to participate in either the qualitative or quantitative phases of the study, as well as alter the relationship between the two actors during the consultation. However, in order to preserve the ethical integrity of the study informed consent had to be obtained, and therefore the minimum amount of information was made available to patients in the waiting room allowing them to decide whether or not to participate. GPs volunteered to take part having been informed as to the nature of the study.

The impact of observers on ethnographic data is a subject much debated and discussed in the social sciences. Nevertheless, it is an inevitable consequence of this type of research. Patients and physicians alike may alter their behaviour in the presence of a third party. In this study, several of the GPs participated regularly in training medical students, and therefore were accustomed to having another person present during consultations. Nevertheless, the aim here is to compare the consultation as experienced by the GP to the consultation as experienced by the patient. Even if their behaviours were altered by the presence of a third party, each actor participated in and experienced the same consultation. Furthermore, the goal of the study was to analyse the interaction between the two participants during the consultation. Thus, whatever the content of the consultation, it is important to analyse the concordance between each actor's understanding of what had happened and was said. Since it is unlikely that the presence of a silent observer should alter the mutual comprehension between two persons, the goal of the study should not be affected in depth by such a presence.

### Data analysis scheme

There are three objectives to the qualitative phase: the first, to verify the feasibility of this type of ethnographic observational study in a general practice setting; the second, to generate new and original results; and the third, to use the qualitative data to develop hypotheses on the interaction between patients and physicians and how the latter may be linked to health inequalities. An inductive sociological approach will be taken using grounded theory analysis whereby the data itself guides the researcher to develop theories [19]. No specific data analysis scheme was set a priori in the aim of allowing the qualitative researchers to develop theories and schemes of analysis as they worked their data. Due to the open and non-prescriptive nature of the data analysis, the researchers will work their material individually, as well as in group sessions, in order to limit the impact of subjectivity on interpreting the data.

Some of the areas that will be explored by the qualitative team will reflect themes present in the literature. The nature of the 'doctor' effect on patient-physician interactions, and therefore the impact of variability between different physicians' manners of working will be an important area to explore. Of particular interest is how GPs might alter their approach to adapt to each individual consultation, taking into account patient characteristics, prior relationships and the medical specificity of each case. With reference to the patient, it will be important to ascertain which factors affect how a patient relates to their GP during the consultation, and whether they feel they have control over their health in general. Differences in the way patients with similar medical conditions are diagnosed and treated will also be an area to explore, more specifically in the context of overweight/obesity being raised during the consultation. Factors that influence the relationship between GPs and patients, and the way in which the consultation may be negotiated between the two parties will be of interest. The notion of a shared identity between physicians and patients as being a key factor in positive health care exchanges, as evoked by Street et al (2008), will be a key theme to explore in both qualitative and quantitative phases of analyses [10].

The quantitative analyses will be developed in more detail based on the hypotheses generated through the qualitative phase when the above themes will be thoroughly explored and new areas of interest identified. The quantitative analyses will be used to compliment the qualitative work, but also to inform it further and suggest new pathways of analysis.

A preliminary quantitative analysis scheme will aim to explore: a) concordances and discordances between the patient and GP about what happened during the consultation, as well as how each party rates the patient's health and whether they were satisfied with the consultation; b) the social context of the consultation by comparing objective and subjective measures of social position as described by the GP versus the patient; c) the relationship between the different social position measures and consultation outcomes, such as treatment, advice and health promotion; d) how gender differences and similarities between GP and patient may affect their interaction, e) the variability in interactions during the consultation and whether this depends primarily on within- or between- GP differences; f) the treatment and follow-up care of obese versus non-obese patients, due to the strong links between obesity and social position.

### First conclusions

The feasibility and positive feedback from patients and GPs alike have been encouraging outcomes thus far observed in the study process. Indeed, both qualitative and quantitative phases were readily accepted by both parties. Patients and GPs were prepared to have a third person present during the consultations, and GPs were willing to accept a qualitative protocol where patients have in-depth post-consultation interviews analysing the nature and type of relationship they have with their doctor. Equally, in the quantitative phase, both parties were willing to fill in a large number of questionnaires, patients being asked to fill-in three separate questionnaires, and GPs having to fill-in a questionnaire for every patient they saw. These aspects that could have posed constraints on the sampling were readily accepted by the participants, and the refusal rate was low.

The development of mirrored semi-structured interviews and mirrored questionnaires in the qualitative and quantitative phases respectively is a methodological success of this study so far. As research tools they will be used to extract valuable data allowing us to compare the patient and GP's perspectives on the consultation, their mutual relationship as well as the patient's social circumstances and their respective assessment of the patient's health status.

Thus far, the multidisciplinary nature of this study has been successful, with all disciplines working independently as well as together to develop the study protocol, collect data and secure funding. Nevertheless the real challenge will be in how well the different parties can collaborate during the analysis phase. Overall, the different disciplines working on the project will develop their respective schemes of analysis together, updating their results and analytical pathways in a circular manner each informing and complimenting the other. A series of workshops will be organised in order to set the work pace, to decide upon important definitions and procedures and to identify common themes. Though no easy feat, this manner of working will maximise the potential of this type of multidisciplinary study and optimise the value of mixed methods research.

To our knowledge this study is original in its design and will contribute to understanding what goes on during a consultation between patients and GPs, and identify what elements of the consultation could contribute to generating health inequalities. Through exploring the interaction between patients and GPs, recommendations can be made to improving primary and secondary health care in France, with the aim of reducing health inequalities and ameliorating the health care system.

## Abbreviations

GP: General practitioner; IF: Ile de France; MP: Midi Pyrenées; PL: Pays de la Loire; BMI: Body mass index (kg/m^2^).

## Competing interests

The authors declare that they have no competing interests.

## Authors' contributions

All authors read and approved the final manuscript. MKI prepared the data and drafted the manuscript. CR, AA, PD, PL, JP and TL contributed to the conception, design, acquisition and interpretation of data, and revised the manuscript.

## Pre-publication history

The pre-publication history for this paper can be accessed here:


